# Molecular Epidemiology of Porcine Cytomegalovirus (PCMV) in Sichuan Province, China: 2010–2012

**DOI:** 10.1371/journal.pone.0064648

**Published:** 2013-06-06

**Authors:** Xiao Liu, Shan Liao, Ling Zhu, Zhiwen Xu, Yuancheng Zhou

**Affiliations:** 1 Animal Biotechnology Center, College of Veterinary Medicine, Sichuan Agricultural University, Ya’ an, China; 2 Key Laboratory of Animal Disease and Human Health, College of Veterinary Medicine, Sichuan Agricultural University, Ya’ an, China; University of Westminster, United Kingdom

## Abstract

Porcine cytomegalovirus (PCMV) is an immunosuppressive virus that mainly inhibits the immune function of the macrophage and T-cell lymphatic systems, and has caused huge economic losses to the porcine breeding industry. Molecular epidemiological investigation of PCMV is important for prevention and treatment, and this study is the first such investigation in Sichuan Province, Southwest China. A PCMV positive infection rate of 84.4% (865/1025) confirmed that PCMV is widely distributed in Sichuan Province. A phylogenetic tree was constructed based on the PCMV glycoprotein B gene (*gB*) nucleotide and amino acid sequences from 24 novel Sichuan isolates and 18 other PCMV *gB* sequences from Genbank. PCMV does not appear to have evolved into different serotypes, and two distinct sequence groups were identified (A and B). However, whether PCMV from this region has evolved into different genotypes requires further research. Analysis of the amino acid sequences confirmed the conservation of *gB*, but amino acid substitutions in the major epitope region have caused antigenic drift, which may have altered the immunogenicity of PCMV.

## Introduction

Porcine cytomegalovirus (PCMV) belongs to the genus *Cytomegalovirus*, subfamily *Betaherpesvirinae*, family *Herpesviridae* [1], and is an icosahedral virus with a double-stranded linear DNA genome. The virus particle diameter is 150–200 nm. PCMV is distributed globally, with reported cases in Britain, Japan, Germany, and the United States [2]. Rapidly progressive consumptive coagulopathy has been observed frequently in swine-to-baboon renal xenotransplantation, and there is also a risk of zoonotic transfer of PCMV in swine-to-human xenotransplantation. However, the risk of PCMV infection for human recipients needs further research [3], [4], [5].

PCMV induces fatal systemic infections in young animals, and can cause death in piglets and embryos. Infected animals show clinical symptoms of inclusion body rhinitis, pneumonia, and dysplasia, as well as disease of the nervous system. Similar to human cytomegalovirus (HCMV) and other cytomegaloviruses, lifelong latent infection often occurs in recovered pigs [6], [7], [8]. PCMV is highly host specific, and is unable to replicate in rabbits, rats, chicken embryos, or in a guinea pig cell culture system. Efforts to study PCMV have been hindered by its specificity for porcine epithelial cells. Cross-infection of PCMV and other viruses often occurs in immunosuppressed swine, and a variety of bacterial infections can occur following PCMV infection [9]. Therefore, PCMV has been recognized as a serious hazard for the pig breeding industry.

Currently, research on PCMV is lacking. Previous work has primarily concentrated on the DNA polymerase (*DPOL*) gene, glycoprotein B (*gB*) gene, and the ORF 40-like protein gene, and sequences for these regions are available in GenBank. Genetic diversity has been noted in the *DPOL* and *gB* genes of different PCMV strains, resulting in antigenic variation; however, there is no evidence to confirm that PCMV has evolved significantly different serotypes or genotypes [9], [10], [11].

The *gB* gene is highly conserved within the *Herpesviridae* family, and is widely used in species identification and phylogenetic analysis of herpesvirus species [9], [12], [13]. Several PCMV sequences from China are available in GenBank, but no systematic genome analysis of Chinese strains has been performed. In this study, we report the detection and characterization of PCMV based on sequencing and phylogenetic analysis of the *gB* nucleotide and amino acid sequences from a large quantity of samples collected from major breeding bases and rural farms in Sichuan Province, China.

## Materials and Methods

### Ethical Statement

Animal welfare standard in this study was established on the basis of internationally agreed and science based principles within the World Organisation for Animal Health (OIE). All experiments were carried out in accordance with China Animal Welfare Legislation and were approved by the Sichuan Agricultural University Committee on Ethics in the Care and Use of Laboratory Animals.

### Samples

**Figure 1:**
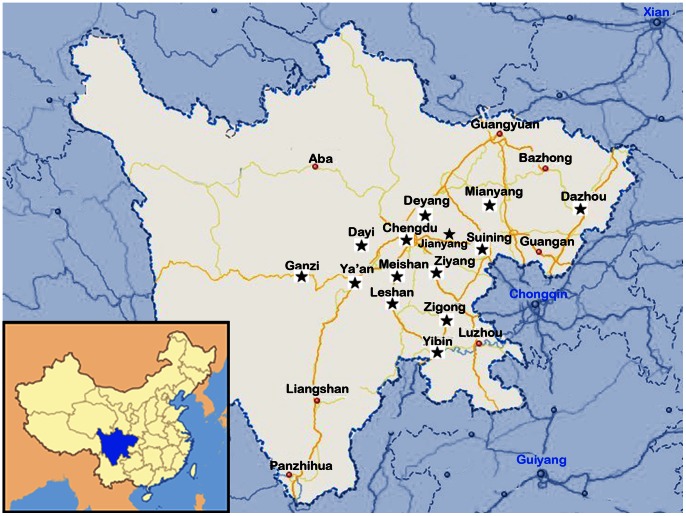
Geographical locations of samples collected in this study. The stars indicate the districts of Sichuan Province where the samples were collected.

A total of 670 porcine serum samples, 322 pulmonary hilar lymph node, spleen, kidney, liver, tonsil, and brain tissue samples, and 33 semen samples were randomly sampled from 1025 piglets, nursery pigs, sows, and boars from major breeding bases and rural farms in different districts of Sichuan Province between August 2010 and July 2012 (Figure 1).

### DNA Extraction

PCMV genomic DNA was extracted from serum, semen, and tissue samples using a commercially available DNA extraction kit (Tiangen Biotech, Beijing, China) according to the manufacturer’s instructions. The extracted DNA was quantitated by SmartSpec Plus Spectrophotometer (Hercules, CA, USA) and stored at −20°C until use in PCR reactions.

### Virus Detection by PCR

The extracted DNA was screened by nested PCR for PCMV DNA using specific primers, P1-1∶5′-CGTGGGTTACTATGCTTCTC-3′, P1-2∶5′-CTTTCTAACGAGTTCTACGC-3′, P2-1∶5′-TGGCTCAGGAAGAGAAAGGAAGTG-3′, and P2-2∶5′-GACGAGAGGACATTGTTGATAAAG-3′, which amplify a 236-bp region of the conserved *DPOL* gene. Amplification was carried out in PCR buffer containing 200 μM of each dNTP, 10 pmol of each primer, 1.0 U *Taq* DNA polymerase (Promega, Madison, WI, USA), and 1.5 mM MgCl_2,_ in a total volume of 25 μL. PCR was performed at 94°C for 3 min, followed by 35 cycles of 94°C for 30 s, 55°C for 30 s, and 72°C for 35 s, and a final extension of 72°C for 8 min. PCR products were electrophoresed on a 1.5% agarose gel, followed by staining with ethidium bromide (Invitrogen, Carlsbad, CA, USA) and visualization under ultraviolet (UV) light on a Bio-Rad gel imaging system (Hercules, CA, USA).

### Amplification of the PCMV *gB* Gene

PCMV genomic DNA was extracted from 24 antigen-positive samples as described above, and the primers P1∶5′-**ATG**ACAGTGAGCAGTCGGAATTTATTCCGGAT-3′ and P2∶5′-**TCA**CACGTCCTCGGTGGATAGCTGC-3′ were used to amplify the complete *gB* gene. The primers sets covered the entire coding region of the PCMV *gB* gene. PCR amplification was carried out as described above. The PCR products were electrophoresed on a 1.5% agarose gel, stained with ethidium bromide, and then visualized under UV light, as above.

### Cloning and Sequencing of the PCMV *gB* Gene

The PCMV *gB* gene amplicons were gel-purified using a Gel Extraction Kit (Tiangen Biotech) and then cloned into pMD19-T Simple Vector (Takara, Dalian, China). The ligated products were transformed into *Escherichia coli* DH5α competent cells (Invitrogen). Colonies containing plasmids with an insert were identified by blue/white selection, and positive colonies were picked and grown in BL medium at 37°C for 8 hours. Plasmid DNA was then extracted using a Plasmid Mini Kit (Omega Bio-Tek, Norcross, GA, USA). Purified plasmid DNA was used as a template for sequencing (Invitrogen, Shanghai, China).

### Phylogenetic Analyses

The amplified PCMV *gB* nucleotide and deduced amino acid sequences were assembled using LaserGene (DNAstar, Madison, WI, USA) and compared with nucleotide and amino acid sequences of 18 PCMV *gB* genes available from GenBank. The sequence homology was confirmed using the Basic Local Alignment Search Tool (BLAST) (http://blast.ncbi.nlm.nih.gov/). Multiple alignment was performed by ClustalW analysis [14], and Molecular Evolutionary Genetics Analysis (MEGA) software version 5.0 was used to conduct phylogenetic analyses and produce the neighbor-joining (NJ) and maximum-likelihood (ML) trees [15]. All analyses were based on the 2583 bp PCMV *gB* gene complete sequences and carried out using the Kimura 2-parameter model [16].

### *gB* Nucleotide and Amino Acid Sequence Analyses

The nonsynonymous to synonymous nucleotide substitution rates were estimated by the Synonymous Nonsynonymous Analysis Program (SNAP) [17]. The transition/transversion bias (*R*) and substitution rates were estimated by MEGA 5.0. The substitution pattern and rates were estimated under the Kimura 2-parameter model, using nucleotide frequencies of A = 25.00%, T/U = 25.00%, C = 25.00%, and G = 25.00%. For estimating ML values, a user-specified topology was used. The maximum Log likelihood values for this computation were −713.948 and −737.102. Codon positions included were 1^st^ +2^nd^ +3^rd^+noncoding. All positions containing gaps and missing data were eliminated, and there were 2580 positions in the final dataset. Evolutionary analyses were conducted in MEGA 5.0 [16], [18]. The Data Analysis in Molecular Biology and Evolution program (DAMBE) was used to establish the plots of transition (s) and transversion (v) frequencies against the K80 distance, and to estimate the amino acid substitution propensity [19]. The gB protein phosphorylation sites were predicted using NetPhos 2.0 [20], analysis of the secondary structure was based on the Network Protein Sequence Analysis server (NPS@) [21], and transmembrane topology was analyzed using the Network server [22]. Three-dimensional (3-D) molecular structures were constructed by the Swiss-Model workspace (alignment mode) [23]. The T cell epitopes were predicted by NetChop 3.1 [24], and the image of the T cell epitopes was generated using Protean (DNAstar).

### Accession Numbers

**Figure 2:**
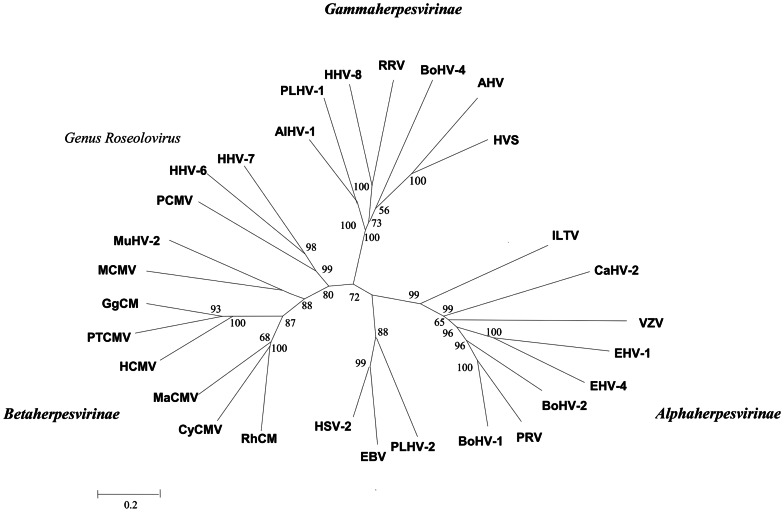
Phylogenetic analysis of *Herpesviridae* viruses. Glycoprotein B nucleotide sequences of the alphaherpesviruses, betaherpesviruses, and gammaherpesviruses were obtained from GenBank: PCMV SC strain (accession no. JN701021.1); RhCMV (Rhesus cytomegalovirus; accession no. U76749.1); CyCMV (Cynomolgus macaque cytomegalovirus; accession no. HQ198248.1); MaCMV (Mycobacteriophage Barnyard; accession no. AY129339); PTCMV (Pan troglodytes cytomegalovirus; accession no. FJ538485); HCMV (Human cytomegalovirus; accession no. M6092.1); GgCMV (Gorilla cytomegalovirus; accession no. FJ538490); MuHV-2 (Murine herpesvirus type 2; accession no. AF232689.2); MCMV (Murine cytomegalovirus; M86302.1); HHV-6 (Human herpesvirus; accession no. M97927.1); PRV (Pseudorabies virus; accession no. AF257079.1; HHV-7 (Human herpesvirus; accession no. AF007830.1); AlHV-1 (Alcelaphine herpesvirus 1; accession no. AF005370); BoHV-1 (Bovine herpesvirus type 1; accession no. Z78205); BoHV-2 (Bovine herpesvirus type 2; accession no. Z78205); BoHV-4 (Bovine herpesvirus type 4; accession no. Z15044.1); EBV (Epstein-Barr virus; accession no. V01555.2); EHV-1 (Equine herpesvirus 1 strain Ab4; accession no. AY665713.1); EHV-4 (Equine herpesvirus 4 strain NS80567; accession no. AF030027.1); HHV-8 (Human herpesvirus 8 type M; accession no. U75698.1); VZV (Varicella-Zoster virus; accession no. X04370); PLHV-2 (porcine lymphotropic herpesvirus 2; accession no. AF191043); PLHV-1 (lymphotropic herpesvirus 1; accession no. AF478169.1); MuHV-2 (Murine herpesvirus 2; accession no. GU018179.1); HSV-2 (Herpes simplex virus 2; accession no. Z86099); AHV (Ateles herpesvirus; accession no. AF083424); ILTV (Laryngotracheitis Virus; accession no. X56093.1).Bootstrap values of **>**65% from 1000 pseudo-replicates are indicated at the branch nodes.

**Figure 3:**
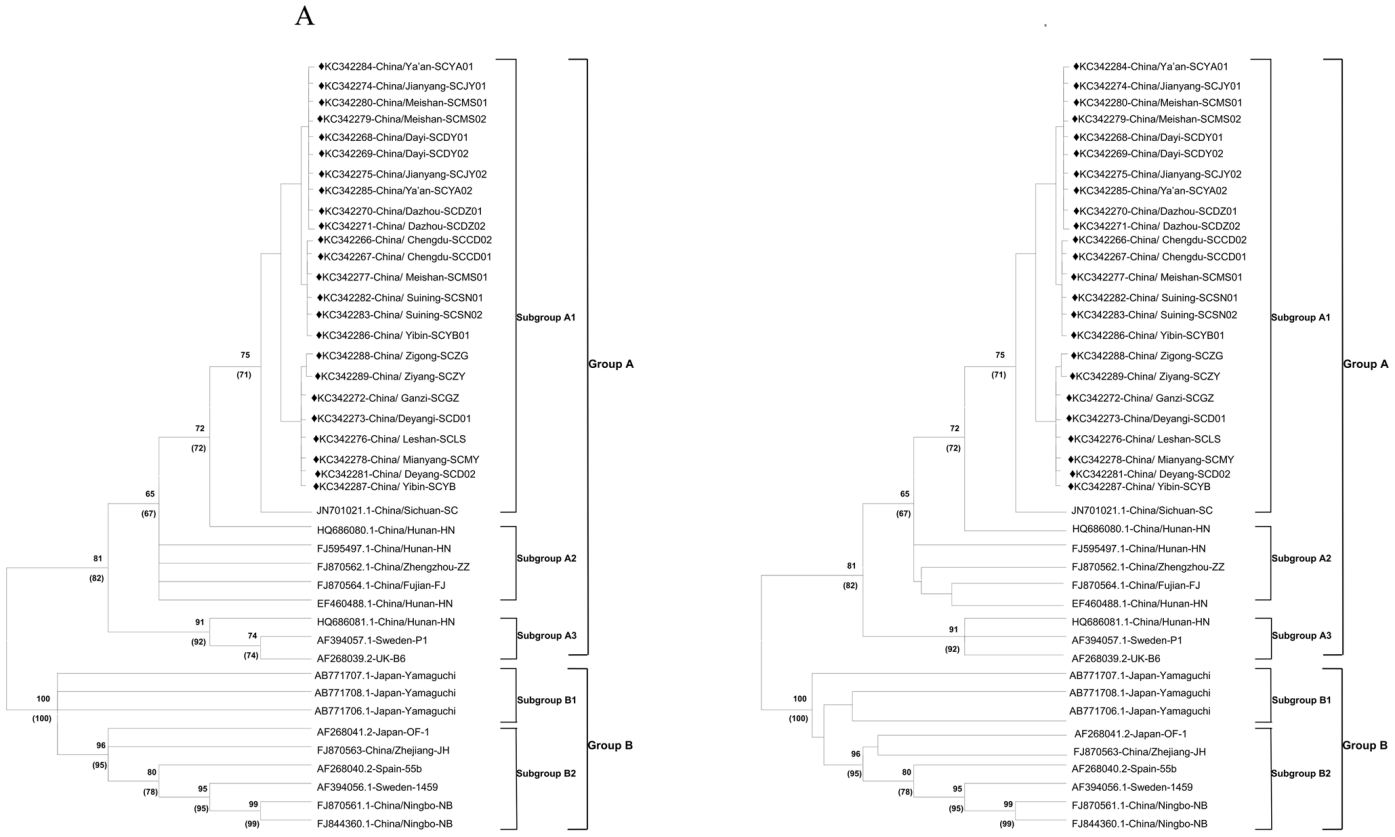
Phylogenetic analysis of 42 global strains of PCMV based on the 2580 bp *gB* complete nucleotide sequence and the deduced amino acid sequence. (A) Phylogenetic tree constructed from nucleotide sequences. (B) Phylogenetic tree constructed from amino acid sequences. The reference PCMV *gB* nucleotide sequences were obtained from GenBank: SC strain (accession no. JN701021.1); ZZ strain (accession no. FJ870562.1); NB strain (accession no. FJ844360.1, FJ870561.1); FJ strain (accession no. FJ870564.1); JH strain (accession no. FJ870563.1); HN isolate (accession no.HQ686081.1, HQ686080.1, FJ595497.1, EF460488.1); Spanish 55b isolate (accession no. AF268040.2); B6 strain (accession no. AF268039.2); Japanese OF-1 strain (accession no. AF268041.2); Japanese Yamaguchi strain (accession no. AB771707.1, AB771708.1, AB771706.1); Swedish isolate P1 (accession no. AF394057.1); Swedish isolate 1469 (accession no. AF394056).Multiple alignment was performed in the Clustal W program, and MEGA 5.0 software was used to construct the neighbor-joining (NJ) and maximum likelihood (ML) trees. Bootstrap values >65% from 1000 pseudo-replicates are indicated at the branch nodes. Numbers in parentheses are the maximum likelihood. Strains are designated by accession number, country/district, and strain name. The 24 strains analyzed in this study are indicated by (♦). The two major groups were identified as A and B.

The novel PCMV *gB* nucleotide sequences have been submitted to NCBI GenBank and assigned accession numbers KC342266–KC342289. Further *Herpesviridae* glycoprotein B nucleotide sequences were obtained from GenBank (http://www.ncbi.nlm.nih.gov/nuccore), Specific accession numbers are given in Figure 2 and Figure 3.

## Results

### PCMV Detection

The collection locations of the 1025 samples were distributed throughout 14 districts in Sichuan Province. Overall, the PCMV positive infection rate was 84.4% (865/1025), and the infection rate in different tissue samples was 91% (323/355). The rate of infection in serum samples was 80.9% (509/637), and was 100% (33/33) in semen samples. There were a further 25 samples that were putatively positive for PCMV but could not be confirmed, and were therefore not included in the positive rates of infection. The PCMV infection rates were greater than 80% in Yibin, Suining, Dazhou, Ya’an, Mianyang, Dayi, Meishan, Zigong, Leshan, and Ganzi districts, with rates of 66.7% (24/36), 60.5% (23/38), 77.3% (75/97), and 76.5% (52/68) detected in Chengdu, Jianyang, Deyang, and Ziyang, respectively. Except in samples collected from Ya’an, cross-infections were detected in all samples from the various districts of Sichuan province (Table 1). The PCMV antigen detection rates at different growth stages of pigs revealed that piglets have the highest PCMV infection rate (91.6%, 163/178), with PCMV infection rates of 80.4% (410/510) and 88% (220/250) in sows and nursery pigs, respectively. Boars showed the lowest rates of infection (46.7%, 42/92).

**Table 1:** PCMV, PRRSV, TTV1, and PRV infection and co-infection rates in different districts of Sichuan Province.

District	Infection rate (%)							
	PCMV	PRRSV	TTV1	PRV	PCMV and PRRSVCo-infection	PCMV and TTV1Co-infection	PCMV and PRVCo-infection	PCMV, PRRSV,TTV1 andPRV Co-infection
Yibin	96.2% (50/52)	23.1% (12/52)	28.8% (15/52)	15.4% (8/52)	23.1% (12/52)	28.8% (15/52)	13.5% (7/52)	3.8% (2/52)
Suining	92.7% (102/110)	37.3% (41/110)	24.5% (27/110)	14.5% (16/110)	36.4% (40/110)	23.6% (26/110)	14.5% (16/110)	0.9% (1/110)
Chengdu	66.7% (24/36)	52.8% (19/36)	19.4% (7/36)	0	52.8% (19/36)	19.4% (7/36)	0	0
Daizhou	97.2% (105/108)	29.6% (32/108)	0	0	29.6% (32/108)	0	0	0
Yaan	100% (20/20)	0	0	0	0	0	0	0
Mianyang	93% (106/114)	46.5% (53/114)	31.6% (36/114)	36% (41/114)	43.9% (50/114)	31.6% (36/114)	34.2% (39/114)	12.3% (14/114)
Dayi	98.6% (73/74)	94.6% (70/74)	59.5% (44/74)	75.7% (56/74)	94.6% (70/74)	59.5% (44/74)	75.7% (56/74)	41.9% (31/74)
Jianyang	60.5% (23/38)	34.2% (13/38)	0	31.6% (12/38)	28.9% (11/38)	0	18.4% (7/38)	15.8% (6/38)
Meishan	94% (94/100)	92% (92/100)	42% (42/100)	84% (84/100)	92% (92/100)	40% (40/100)	84% (84/100)	38% (38/100)
Deyang	77.3% (75/97)	48.5% (47/97)	59.8% (58/97)	33% (32/97)	37.1% (36/97)	49.5% (48/97)	27.8% (27/97)	22.7% (22/97)
Zigong	92.5% (37/40)	0	15% (6/40)	0	0	15% (6/40)	0	0
Leshan	90% (54/60)	58.3% (35/60)	21.7% (13/60)	21.7% (13/60)	28.3% (17/60)	13.3% (8/60)	10% (6/60)	66.7% (4/60)
Ziyang	76.5% (52/68)	38.2% (26/68)	25% (17/68)	50% (34/68)	19.1% (13/68)	7.4% (5/68)	20.6% (14/68)	5.9% (4/68)
Ganzi	82.7% (62/75)	0	0	13.3% (10/75)	0	0	10.7% (8/75)	0

The PRRSV, TTV1, PRV infection of all samples has been detected by PCR (670 serum +322 tissue).

### PCR Amplification, Sequence Alignment, and Phylogenic Analysis

PCRs performed with primers P1 and P2 amplified the expected 2580 bp complete PCMV *gB* gene fragment. The authenticity of the fragments cloned into the pMD19-T Simple Vector was confirmed by agarose gel electrophoresis and DNA sequencing. The 24 nucleotide and deduced amino acid sequences of the PCMV *gB* genes obtained from samples from 14 different districts of Sichuan Province were used to construct phylogenetic trees using the neighbor joining (NJ) and maximum likelihood (ML) methods. The nucleotide and amino acid sequence data of 18 PCMV and 29 *Herpesviridae* reference strains obtained from the GenBank database were used for comparison. Bootstrap values (>60) were obtained for both the NJ and ML trees.

Phylogenetic analysis of the *Herpesviridae* viral samples used in this study confirmed that PCMV strain SC (accession no. JN701021.1) is clustered with human herpes virus type 6 (HHV-6) and human herpes virus type 7 (HHV-7). PCMV strain SC, HHV-6, and HHV-7 were shown to belong to the same evolutionary branch of the subfamily *Betaherpesvirinae* (Figure 2), with nucleotide homologies of 48.5% and 49.4% for HHV-6 and HHV-7 *gB* sequences, respectively. PCMV showed less homology to Rhesus cytomegalovirus (RhCMV), *Cynomolgus macaque* cytomegalovirus (CyCMV), *Pan troglodytes* cytomegalovirus (PtCMV), murine cytomegalovirus (MCMV), gorilla cytomegalovirus (GgCMV), Mycobacteriophage Barnyard cytomegalovirus (MaCMV), and human cytomegalovirus (HCMV), with homologies 13.3–43.8%.

The phylogenetic and homology analysis results at the nucleotide (2580 bp) and amino acid (860 aa) levels showed that global PCMV strains belonged to two distinct sequence groups (A and B), which were separated with bootstrap values of 81 and 100, respectively. The PCMV *gB* gene sequences determined in this study, the 24 novel PCMV strains, the SC strain (accession no. JN701021.1), the ZZ strain (accession no. FJ870562.1), the NB strain (accession no. FJ844360.1, FJ870561.1), the FJ strain (accession no. FJ870564.1), the JH strain (accession no. FJ870563.1), the HN isolate (accession no.HQ686081.1, HQ686080.1, FJ595497.1, EF460488.1) the B6 strain from the United Kingdom (accession no. AF268039.2), and Swedish isolate P1 (accession no. AF394057.1) all belonged to A group, while isolate 55b from Spain (accession no. AF268040.2), strain OF-1 from Japan (accession no. AF268041.2), isolate 1469 from Sweden (accession no. AF394056), the JH strain (accession no. FJ870563.1), and the NB strain (accession no. FJ844360.1, FJ870561.1) belonged to B group (Figure 3). The 24 PCMV sequences identified in this study belong to the SC strain, and the nucleotide homology of the 24 *gB* gene sequences was 99.4–100%, with deduced amino acid sequence homologies of 98**–**100%. The nucleotide homology between the global PCMV strains and the newly obtained PCMV strains was 98–99%, with homologies of the deduced amino acid sequences of 97–99%.

### Glycoprotein B Sequence Analyses

**Figure 4:**
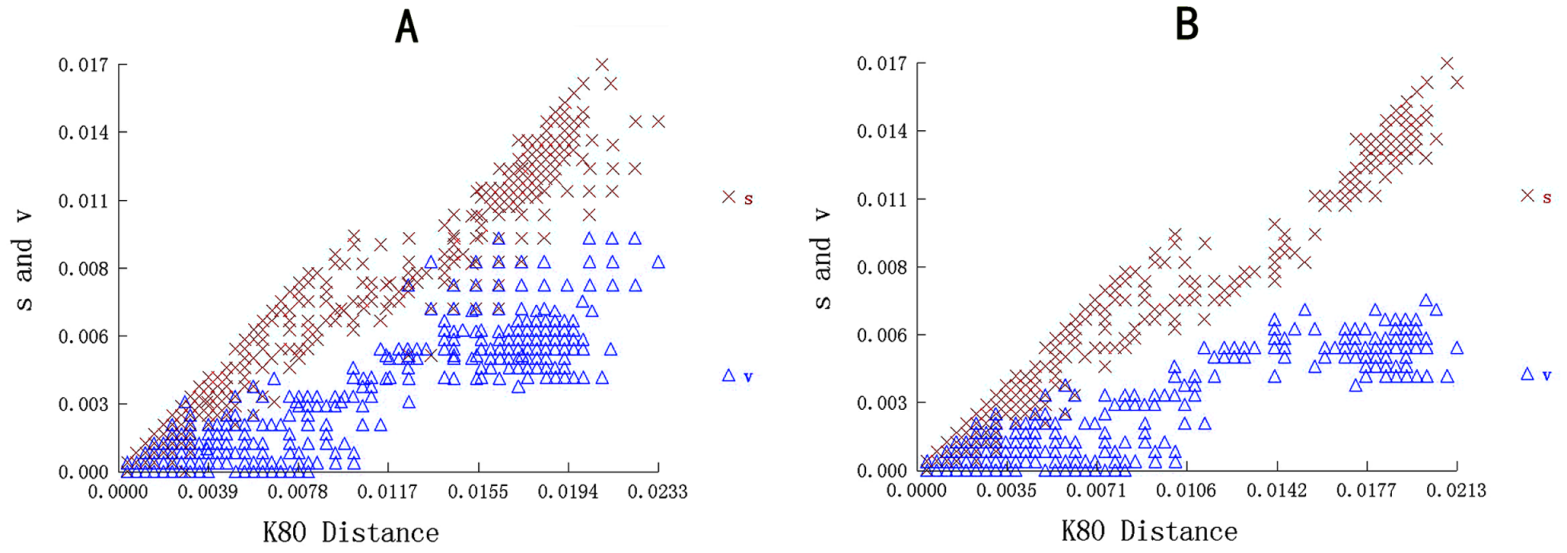
Plots of transition (s) and transversion (v) frequencies against the K80 distance. *S* denotes transition and *V* denotes transversion. (A) Analysis of 34 PCMV strains from China. (B) Analysis of global PCMV strains.

The 24 novel *gB* nucleotide sequences and their deduced amino acid sequences were also aligned. The maximum Kimura distance between the PCMV *gB* nucleotide sequences of the 24 novel Sichuan strains and the other global strains was 2.33%, in comparison with 2.13% between the *gB* nucleotide sequences of ten Chinese PCMV strains (Figure 4). The estimated transition/transversion bias (*R*) was 8.03 among the 34 PCMV strains from China, and 6.03 among global PCMV strains. The newly obtained Sichuan PCMV nucleotide sequences presented 30 common mutations (at nucleotide positions 82, 83, 85, 97, 124, 138, 308, 351, 369, 428, 457, 589, 735, 868, 1235, 1428, 1472, 1565, 1566, 1685, 2175, 2363, 2372, 2375, 2376, 2433, 2434, 2471, 2503, 2506) in the *gB* nucleotide sequence, 22 of which were found in more than one sequence. Based on a set of codon-aligned *gB* nucleotide sequences, the dN/dS (nonsynonymous to synonymous substitution) rates among the global and Sichuan strains were 2.39 (0.82/0.6) and 1.82 (0.39/0.27) respectively. The maximum likelihood estimates of the substitution matrix were also calculated (Table 2).

**Table 2:** Maximum likelihood estimate of substitution matrix.

34 PCMV strains from China					Global PCMV strains				
	A	T/U	C	G		A	T/U	C	G
**A**	–	*1.39*	*1.39*	**22.23**	**A**	–	*1.78*	*1.78*	**21.44**
**T/U**	*1.39*	–	**22.23**	*1.39*	**T/U**	*1.78*	–	**21.44**	*1.78*
**C**	*1.39*	**22.23**	–	*1.39*	**C**	*1.78*	**21.44**	–	*1.78*
**G**	**22.23**	*1.39*	*1.39*	–	**G**	**21.44**	*1.78*	*1.78*	–

Analysis of 34 PCMV strains from China and global PCMV strains. Substitution pattern and rates were estimated under the Kimura 2-parameter model. Rates of different transition substitutions are shown in **bold** and those of transversion substitutions are shown in *italics*.

**Figure 5:**
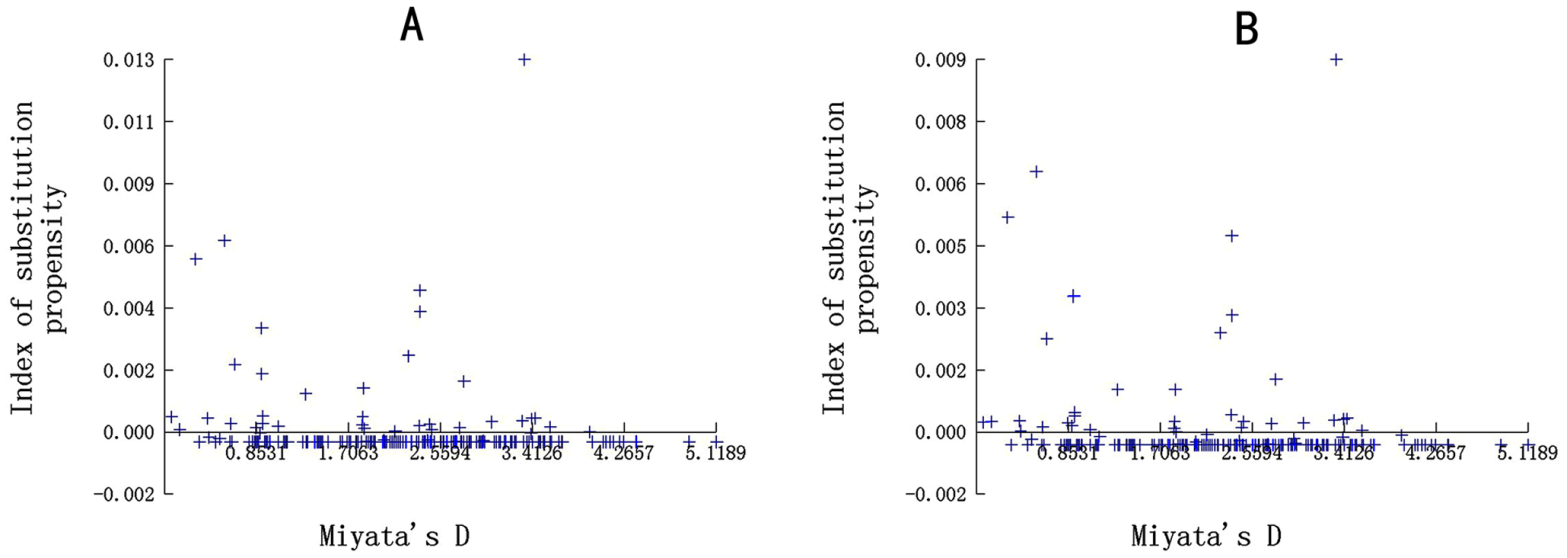
Index of amino acid substitution propensity. (A) Analysis of 34 PCMV strains from China. (B) Analysis of global PCMV strains.

The 18 deduced glycoprotein B amino acid sequences from the global strains were aligned and analyzed along with the novel 24 Sichuan strains. The maximum index of substitution propensity was 0.9% between the Spanish 55b strain (accession no. AF268040.2), the B6 strain (accession no. AF268039.2), the Japanese OF-1 strain (accession no. AF268041.2), the Japanese Yamaguchi strain (accession no. AB771707.1, AB771708.1, AB771706.1), and the Swedish strain (accession no. AF394056, AF394057.1), in comparison with 1.3%% between the SC strain (accession no. JN701021.1), the ZZ strain (accession no. FJ870562.1), the NB strain (accession no. FJ844360.1, FJ870561.1), the JH strain (accession no. FJ870563.1), the FJ strain (accession no. FJ870564.1), and the HN isolates (accession no.HQ686081.1, HQ686080.1, FJ595497.1, EF460488.1) (Figure 5).

**Figure 6:**
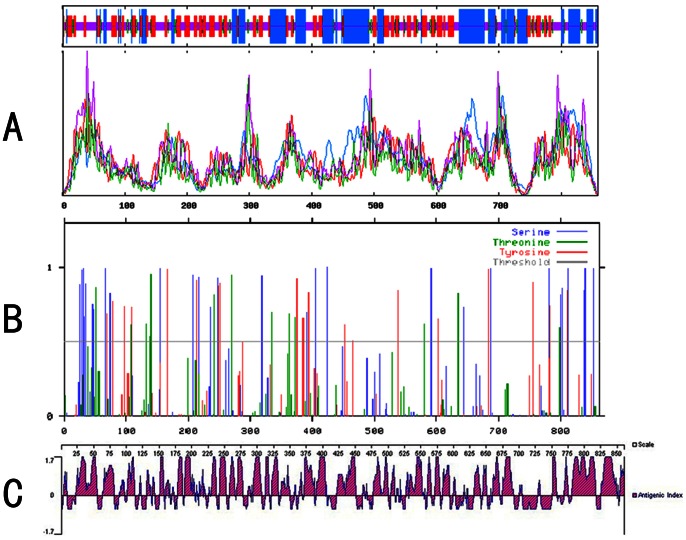
Illustration and sequence alignment of the gB protein. (A) Analysis of the secondary structure. The α-helix, extended strand, loop region, and β-turn are indicated by blue, red, yellow, and green curves, respectively. (B) Phosphorylation site prediction. (C) Prediction of T-cell epitopes.

**Figure 7:**
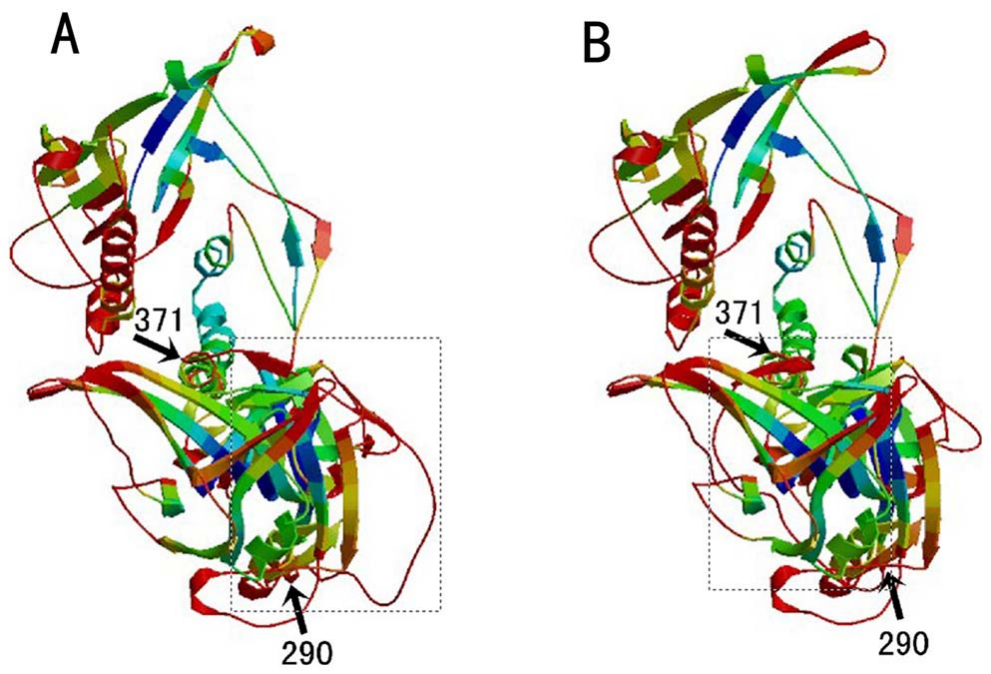
Three-dimensional structure prediction for alignment of the gB sequence of two viral strains, (A) JN701021.1-China-SC, (B) AF268040.2-Spain-55b. The loop region (random coil) of the gB protein is shown in the closed box, and amino acid residues 290–371 is indicated.

Among the global PCMV strains, Most amino acid substitutions were concentrated at the C–terminus, the β–turn, and the loop region (random coil) of the gB protein (Figure 6A). Prediction of the phosphorylation sites in the gB amino acid sequence showed there are 51 potential phosphorylation sites with a threshold value of 0.5, including 29 serine phosphorylation sites, 13 threonine phosphorylation sites, and 19 tyrosine phosphorylation sites (Figure 6B). The T-cell epitopes were mainly distributed at positions 23–109 aa and 769–861 aa of the gB protein (Figure 6C). The Sichuan strains had 15 common amino acid substitutions (T^28^→Y, P^42^→A, D^104^→N, S^153^→P, N^197^→Y, G^245^→D, T^290^→A, G^412^→A, E^491^→G, E^791^→A, F^792^→S, Y^812^→D, I^824^→T, N^835^→D, A^836^→T) (Table 3), including eight phosphorylation site substitutions. The 3-D conformation prediction showed that the substitutions in the loop region and β-turn altered not only the conformation of these regions, but also altered the conformation of the neighboring regions (Figure 7).

**Table 3:** Data from the 24 PCMV clinical isolates and prototype Sichuan strain (GenBank accession no. JN701021.1).

District	Designation	Specimen	Category	Amino acid substitutions													
				28	42	104	153	197	245	290	412	491	791	792	812	824	836
Chengdu	KC342266- SCCD01	Hepar	nursery swine	–	P	N	–	–	D	A	A	G	–	S	–	–	–
	KC342267- SCCD02	Hepar	nursery swine	Y	P	N	–	–	D	A	A	G	–	S	–	–	–
Dayi	KC342268- SCDY01	Pulmonary hilar lymph node	piglet	–	P	N	P	–	D	A	–	G	A	–	D	–	–
	KC342269- SCDY02	Pulmonary hilar lymph node	piglet	–	P	N	P	–	D	A	–	G	A	–	–	–	–
Daizhou	KC342270- SCDZ01	Hepar	nursery swine	–	P	N	–	–	D	A	–	G	–	–	–	–	A
	KC342271- SCDZ02	Hepar	nursery swine	–	P	N	–	–	D	A	–	G	–	–	–	–	–
Ganzi	KC342272- SCGZ	Spleen	sow	–	P	N	–	–	D	A	–	G	–	–	–	–	A
Deyang	KC342273- SCD01	Hepar	piglet	–	P	N	–	–	D	A	–	G	–	–	–	–	A
	KC342281- SCD02	Hepar	piglet	–	P	N	–	–	D	A	–	G	–	–	–	–	A
Jianyang	KC342274- SCJY01	Hepar	sow	–	P	N	P	–	D	A	–	G	–	–	–	T	–
	KC342275- SCJY02	Hepar	sow	–	P	N	P	–	D	A	–	G	–	–	–	T	–
Leshan	KC342276- SCLS01	Pulmonary hilar lymph node	piglet	–	P	N	–	–	D	A	–	G	–	–	–	–	A
Mianyang	KC342277- SCMY01	Pulmonary hilar lymph node	sow	–	–	N	–	–	D	A	–	G	–	–	–	–	A
	KC342278- SCMY02	Pulmonary hilar lymph node	sow	–	–	N	–	–	D	A	–	G	–	–	–	–	–
Meishan	KC342279- SCMS01	Hepar	sow	–	P	N	P	Y	D	A	–	G	–	–	–	–	–
	KC342280- SCMS02	Hepar	sow	–	P	N	P	–	D	A	–	G	–	–	–	–	–
Suining	KC342282- SCSN01	kidney	piglet	–	A	N	P	Y	D	A	–	G	–	–	–	–	–
	KC342283- SCSN02	kidney	piglet	–	A	N	P	–	D	A	–	G	–	–	–	–	–
Yaan	KC342284- SCYA01	Pulmonary hilar lymph node	piglet	Y	–	N	P	Y	D	A	–	G	–	–	–	–	–
	KC342285- SCYA02	Pulmonary hilar lymph node	piglet	–	–	N	P	–	D	A	–	G	–	–	–	–	–
Yibin	KC342286- SCYB01	kidney	piglet	Y	–	N	–	–	D	A	–	G	–	–	–	–	A
	KC342287- SCYB02	kidney	piglet	–	.P	N	–	–	D	A	–	G	–	–	–	–	–
Zigong	KC342288- SCZG	Pulmonary hilar lymph node	piglet	–	P	N	–	–	D	A	–	G	–	–	–	–	A
Ziyang	KC342289- SCZY	Spleen	sow	–	P	N	–	–	D	A	–	G	–	–	–	–	A
	SC JN701021.1			–	P	N	–	–	D	A	–	G	–	–	–	–	–

Strains are designated according to accession number and strain name.

## Discussion

PCMV is an economically important pathogen due to its immunosuppressive effect of inhibiting the immune function of T lymphocytes and macrophages, and PCMV-infected pigs are easily infected by other pathogens [2], [6], [25], [26]. To survey the prevalence of PCMV in Sichuan Province, China, and provide a foundation for its prevention, a large number of serum, semen, and tissues samples were collected from 14 different districts of the province. The sampling area covered the entire territory of Sichuan Province, including the Chengdu Plain and mountain region of Ganzi Prefecture. The PCMV infection in different tissues of the same pig has not been detected in this study, therefore, the distribution regulation of PCMV in animal body need further research.

Nested PCR showed that PCMV infection was widespread in large-scale breeding bases and rural farms of Sichuan, and the antigen positive rate of 84.4% was close to the reported domestic and foreign rates of infection. According to the PCMV infection rates at different growth stages, piglets, nursery pigs, and expectant sows were more vulnerable to PCMV than boars. In this study, we could infer from clinical testing data that vertical transmission was the major transmission mode of PCMV. PCMV antigen detection results from different samples showed the virus is widely distributed in various organs of the body, and mainly affects the immune and respiratory systems. In this study, PCMV was detected for the first time in boar semen. The rate of PCMV infection in sows from the same farm was very high; therefore, we speculate that PCMV may spread through the reproductive system, and it may be necessary to adopt quarantine measures for infected boars. Based on the results of clinical testing, PCMV-infected pigs were always co-infected with porcine reproductive and respiratory syndrome virus (PRRSV), transfusion transmitted virus (TTV1), and pseudorabies virus (PRV). In addition, *Streptococcus*, *Haemophilus parasuis*, *Actinobacillus*, and *Pasteurella suis* infections often occur following PCMV infection, making prevention and treatment more difficult. Therefore, the collaborative immune suppression mechanisms between PCMV and other immunosuppressive viruses are worthy of further study, and we believe that this avenue has great significance for prevention and control of immunosuppressive viruses.

PCMV glycoprotein B is an important transmembrane glycoprotein that plays a major role in the fusion and adhesion of the virus onto the membrane of host cells when the virus enters cells or transfers between cells [27]. Glycoprotein B also plays an important role in the induction of humoral and cellular immune responses. It is the target of cytotoxic T lymphocytes, and is recognized by natural killer cells that have been activated by lymphokine. Glycoprotein B induces protective immunity and has significant immunogenicity, therefore, further research could provide a new approach for the treatment and prevention of PCMV [28], [29]. Molecular epidemiological analysis of PCMV based on full-length *gB* gene sequences from different strains and further gene molecular characterization analyses would help to devise a suitable strategy to prevent losses in production.

This study is the first molecular analysis of the full-length PCMV *gB* nucleotide sequence from PCMV strains isolated worldwide. The nucleotide and amino acid sequences of 24 new Sichuan strains and 13 global strains were aligned and analysed. Phylogenetic tree and homology analysis showed there was no significant variation between *gB* nucleotide and amino acid sequences from different PCMV strains, confirming that the *gB* gene sequences from Sichuan and global PCMV strains are highly conserved, and that the viral genome is stable. These factors are conducive to the development of measures for the prevention and treatment of the virus.

We determined that the homologies of *gB* nucleotide and amino acid sequences investigated in this study were higher than 98% and 97%, respectively. According to the “75%/85% rule”, if more than 75% of the nucleotide sequence or more than 85% of the amino acid sequence is homologous, the sequences are considered to be from the same serotype [30], [31]. This finding confirmed that globally, PCMV strains have not evolved into significantly different serotypes since PCMV was first isolated in 1955 in Britain.

We determined that tree strains from China, the Ningbo strain (accession no. FJ844360.1, FJ870561.1), the Zhejiang strain JH (accession no.FJ870563), isolate 55b from Spain (accession no. AF268040.2), Japanese strain OF-1 (accession no. AF268041.2), the Japanese Yamaguchi strain (accession no. AB771707.1, AB771708.1, AB771706.1), and a Swedish strain (accession no. AF394056.1) belonged to group B. Therefore, we hypothesized that the tree Chinese PCMV strains may have come from Europe or Japan. However, the prevalence of foreign strains was limited to the eastern part of China.

Phosphorylation may affect the stability of the PCMV virus complex. The level of phosphorylation may adjust the binding energy between the viral proteins in the replication complex, and affect the interaction between the viral and host proteins. The results of 3-D conformation prediction showed that the phosphorylation site substitutions of serine, threonine, and tyrosine, at T^28^→Y, S^153^→P, N^197^→Y, T^290^→A, F^792^→S, Y^812^→D, I^824^→T, and A^836^→T (Figure 7), have likely altered the conformation of the PCMV gB protein. These amino acid substitutions were concentrated in the gB major epitope region in PCMV SC strains, and antigenic drift was affected by accumulation of point mutations among the surface proteins. Mutations in the epitope regions may change the tissue tropism of the virus, therefore, the conformational change in the β-turn and loop regions may have already lead to antigenic drift, changed the immunogenicity of the virus to the host, and affected the specific immune response in the organism. However, whether these changes have altered the capacity of gB for adhesion and penetration of the host cell membrane needs further investigation.

## Conclusion

This is the first molecular epidemiological investigation of PCMV in Sichuan Province, China. The PCMV infection rate of 84.4% (865/1025) confirmed that PCMV has been widely distributed in southwest China. PCMV was detected for the first time in porcine semen, allowing us to conclude that it may spread through the reproductive system. The results of nucleotide and amino acid sequence analyses confirmed that the PCMV gB protein is conserved, but that amino acid substitutions in the gB epitope region may cause antigenic drift, and alter the immunogenicity of PCMV.

## Ethical Approval

Animal welfare standard in this study was established on the basis of internationally agreed and science based principles within the World Organisation for Animal Health (OIE). All experiments were carried out in accordance with China Animal Welfare Legislation and were approved by the Sichuan Agricultural University Committee on Ethics in the Care and Use of Laboratory Animals.
